# Additional Role of Myocardial Work in Prognostic Stratification of Patients with Severe Aortic Regurgitation Undergoing Aortic Valve Surgery

**DOI:** 10.3390/diagnostics16111655

**Published:** 2026-05-27

**Authors:** Giulia Elena Mandoli, Gerardo Elia Del Vecchio, Nicolò Ghionzoli, Luca Corda, Pamela Tartaglia, Andrea Stefanini, Maria Concetta Pastore, Francesco Morrone, Marta Focardi, Matteo Lisi, Antonello D’Andrea, Matteo Cameli

**Affiliations:** 1 Department of Medical Biotechnologies, Division of Cardiology, Ospedale “S. Maria alle Scotte”, University of Siena, Viale Mario Bracci, 16, 53100 Siena, Italy; giulia.mandoli@unisi.it (G.E.M.); nicologhionzoli@gmail.com (N.G.); lucacorda96@gmail.com (L.C.); tartagliapamela@gmail.com (P.T.); astefanini94@gmail.com (A.S.); mariaconce.pastore@unisi.it (M.C.P.); fmorrone35@gmail.com (F.M.); focardim@unisi.it (M.F.); matteo.cameli@unisi.it (M.C.); 2 Department of Cardiology, Division of Cardiology and Intensive Care Unit, Ospedale del Mare, ASL Napoli 1 Centro, Via Enrico Russo 11, 80147 Napoli, Italy; 3 Department of Cardiovascular Disease, Division of Cardiology, Ospedale “S. Maria delle Croci”, AUSL Romagna, Viale Randi 5, 48121 Ravenna, Italy; matteo.lisi@hotmail.it; 4 Department of Cardiology, Division of Cardiology and Intensive Care Unit, Ospedale “Umberto I”, ASL Salerno, Via Alfonso de Nicola, 84014 Nocera Inferiore, Italy; antonellodandrea@libero.it

**Keywords:** aortic regurgitation, myocardial work, global work efficiency, speckle-tracking echocardiography, valvular heart disease

## Abstract

**Background/Objectives:** The optimal timing for surgery in severe aortic regurgitation (AR) remains challenging. Current recommendations are based on symptoms, LV size and ejection fraction (LVEF), yet subclinical dysfunction may occur earlier. Myocardial work (MW), derived from LV longitudinal strain (GLS) and systemic systolic arterial pressure, provides a sensitive load-adjusted assessment of myocardial performance. The aim of this study was to evaluate the prognostic role of preoperative MW indices in patients with severe AR undergoing cardiac surgery. **Methods:** This retrospective, single-center observational study included 83 consecutive patients with severe AR referred for surgery. All patients underwent preoperative echocardiography with speckle-tracking and MW analysis. The primary composite endpoint was all-cause mortality or unplanned hospitalization for heart failure. Median follow-up was 25 months. **Results:** Compared with reference values, our patients showed significantly reduced global work index (GWI) (1580 ± 568 vs. 1896 ± 308 mmHg%, *p* < 0.001) and global work efficiency (GWE) (86% vs. 96%, *p* < 0.001) and markedly increased global wasted work (GWW) (328 ± 182 vs. 78.5 ± 51.26 mmHg%, *p* < 0.001), reflecting impaired myocardial efficiency. Patients reaching the primary endpoint exhibited significantly higher GWW (432 ± 224 vs. 295 ± 154 mmHg%, *p* = 0.017) and lower GWE (82% vs. 88%, *p* = 0.013). On univariate analysis, higher GWE was associated with a lower risk of the composite endpoint (HR 0.939, 95% CI 0.888–0.993, *p* = 0.028). A GWE cut-off value of 85.5% provided optimal prognostic stratification, with higher values associated with improved event-free survival. The accuracy for outcome prediction of GWE increased when combined with other echocardiographic parameters, such as LVEF. **Conclusions:** In patients with severe AR, MW analysis—particularly GWE—offers incremental prognostic value, allowing the identification of subclinical ventricular dysfunction. This could lead to better risk stratification and earlier surgical intervention, particularly when combined with other conventional echocardiographic parameters.

## 1. Introduction

Aortic valve diseases are a significant contributor to cardiovascular morbidity and mortality worldwide, accounting for 61% of all valvular heart disease (VHD) deaths [1]. Aortic regurgitation (AR) is considered the fourth most common valvular disease worldwide, and could result from dysfunction of the aortic valve due to abnormalities in the aortic root or proximal ascending aorta, or from primary lesions affecting the valve itself. The prevalence in the general population is estimated at around 4.9% for moderate AR and around 0.5% for severe AR [2,3,4].

In AR, the main pathophysiological mechanism is volume overload, prompting various adaptive mechanisms in the LV [3,5]. These adaptations can vary in acute and chronic AR. Acute AR causes sudden volume overload in a non-compliant left ventricle, increasing LV end-diastolic pressure, inducing pulmonary congestion, and edema.

Reduced effective cardiac output leads to systemic hypoperfusion, impaired coronary perfusion, and ultimately cardiogenic shock [3,5]. In contrast, chronic AR progresses slowly, allowing LV remodeling with dilation, increased compliance, and eccentric hypertrophy that preserves stroke volume despite combined volume and pressure overload. Thus, LV ejection fraction (LVEF) and end-diastolic pressures typically remain normal at the beginning. However, progressive interstitial myocardial fibrosis reduces LV compliance, leading to elevated diastolic pressures and eventual deterioration in LVEF, along with the onset of HF symptoms, which occur belatedly [2,3,5].

The clinical progression of aortic regurgitation (AR) is closely linked to its underlying etiology. For instance, in BAV patients, AR is often complicated by a concomitant aortopathy characterized by aortic root dilatation, which further exacerbates the regurgitant volume through a loss of commissural support. Pathophysiologically, these individuals typically present at a younger age compared to those with degenerative or senile AR. This demographic difference is reflected in a distinct pattern of myocardial remodeling; while degenerative AR often involves chronic pressure-volume overload in an aging myocardium, BAV-associated AR occurs in a setting of potentially different adaptive mechanisms and vascular stiffness. Understanding these divergent pathophysiological frameworks is essential for appropriate non-invasive evaluation and management [6].

Therefore, when left untreated, AR may progress to HF even when completely asymptomatic, underscoring the necessity for timely detection and surgical or percutaneous intervention. While aortic valve replacement or repair offers a path to improved patient outcomes, it is increasingly recognized that preoperative myocardial function can influence postoperative recovery and long-term prognosis, since irreversible myocardial damage could not improve, even after successful AV surgery [5]. Thus, early markers of significant hemodynamic impact on LV are pivotal in the management of AR.

Decisions regarding intervention are primarily guided by the severity of the valve disease and the presence of symptoms, with LVEF playing a larger role in cases where patients are asymptomatic, according to international guidelines. Particularly aortic valve surgery is indicated in asymptomatic patients with severe AR with LVEF < 50% or with dilated LV (LV end systolic diameter > 50 mm or 25 mm/mq after indexing for BSA) [7] However, as already mentioned, adaptative mechanisms contribute to keeping LVEF within normal range until advanced stage. Nonetheless, subclinical LV systolic dysfunction occurs significantly earlier, even in completely asymptomatic patients.

Speckle-tracking echocardiography (STE) offers a potential solution to many of these limitations and enables the measurement of myocardial deformation. Among these, global longitudinal strain (GLS) is the most used in clinical practice to characterize LV systolic function, representing a sensitive and reproducible indicator of LV dysfunction, due to the early detection of impairment in the longitudinal aligned subendocardial LV fibers [8,9,10,11]. The diagnostic and prognostic value of GLS has been largely proven in AR [10,12,13]. However, GLS is influenced by loading conditions, making it difficult to distinguish between abnormal GLS due to intrinsic reduced LV contractility from changes caused by altered load [14].

Myocardial work (MW) represents a novel echocardiographic parameter that could potentially overcome the afterload dependent limitation of GLS, integrating strain analysis with LV pressure estimated non-invasively from peripheral systolic blood pressure (BP). Hence, it offers an enhanced view of myocardial performance in the context of different loading conditions, such as those imposed by aortic valve disease [15,16]. Emerging evidence suggests that MW assessment may be useful in the context of VHD evaluation and management, offering prognostic insights into cardiac surgical outcomes, particularly in predicting ventricular recovery and overall functional improvement [15,16,17].

Despite these insights, there is a scarcity of data examining MW in patients with AR. This retrospective study aims to bridge this gap by evaluating the role of MW in patients with severe AR who have undergone cardiac surgery. Our objective is to elucidate how severe AR could affect MW indices and investigate the relationship between MW indices and postoperative outcomes, thereby advancing our understanding of myocardial function in the context of AR physiopathology and providing a basis for potential risk stratification and management optimization in this population.

## 2. Materials and Methods

### 2.1. Study Population

In this observational and monocentric cohort study, consecutive patients ages > 18 years with AR who had undergone cardiac surgery in an Italian University Hospital, between January 2020 and July 2024, were retrospectively screened. The inclusion criteria were presence of severe AR; pre-operative echocardiography availability with adequate acoustic windows for STE; and brachial artery cuff pressure measured at the time of the exam for MW analysis. Severity of AR was determined according to the American Society of Echocardiography and European Association of Cardiovascular Imaging recommendations [18,19]. Patients were referred for intervention according to the European Society of Cardiology recommendation for management of VHD [7]. Patients with more than mild associated VHD, active infective endocarditis (IE); acute aortic syndromes, prosthetic valve or ring and atrial fibrillation during examination were excluded. The study was performed in accordance with the Declaration of Helsinki and was approved by the local Ethics Committee.

### 2.2. Data Collection and Standard Echocardiographic Examination

Clinical and demographic data were collected using the institution’s electronic records. All echocardiographic examinations, measures and VHDs quantifications were performed by experienced operators using a GE Vivid E80/E95/iq (GE Medical Systems, Lisburn, Northen Ireland) equipped with an adult 1.5–4.3 MHz phased-array transducer, according to the American Society of Echocardiography and European Association of Cardiovascular Imaging recommendations [20,21].

### 2.3. Speckle Tracking and Myocardial Work Analysis

For STE analysis, all apical view segments (four-chamber, two-chamber and apical long-axis) required clear visualization of the endocardial borders and myocardium throughout the entire cardiac cycle. Care was taken to avoid foreshortening of LV and LA. The frame rate was 60–80 frames/s.

LV strain was conducted semi-automatically by software across the three apical views, with adjustments by the operator for precise endomyocardial tracking. If any wall segment was not automatically recognized, the software alerted the operator, who could then manually adjust the region of interest and proceed with the analysis. Whether certain segments could not be adequately tracked, strain was calculated by averaging the values from the remaining tracked segments [9].

For the subsequent MW analysis, markers indicating the opening and closing of the aortic and mitral valves were set visually from the apical long-axis view to define each major phase of the cardiac cycle. Additionally, brachial cuff blood pressure, taken at the time of the echocardiographic exam, was used to warp the reference curve for LV pressure estimation in time and amplitude [15,17,22,23]. The MW indices collected include: the GWI, representing the total work performed by the left ventricle during mechanical systole (from mitral valve closure to mitral valve opening), including isovolumetric contraction and relaxation. GCW, which is the productive work performed during systolic shortening combined with the work done during lengthening in isovolumetric relaxation. GWW, the non-productive work expended during systolic lengthening and shortening in isovolumetric relaxation. Finally, GWE, which is calculated as the ratio between constructive work and the sum of constructive and wasted work (GCW/[GCW + GWW]), is expressed as a percentage [23,24,25,26].

STE and MW analysis was performed off-line using EchoPAC software v204 (GE Medical, Milwaukee, WI, USA) by a single experienced and independent operator, not directly involved in the image acquisition, to reduce biases.

In our analysis MW indices found in the study population were compared to the normal reference values recommended by international societies derived from the NORRE study [25].

### 2.4. Outcomes

Information on outcomes was obtained directly from patients or their relatives by telephone interviews and from electronic records, which were used to determine survival status at the most recent follow-up and the need for unplanned hospitalization for HF. The primary endpoint was a composite of all-cause mortality and unplanned hospitalization for HF. Secondary endpoints included each of these as individual outcomes—all-cause mortality and unplanned hospitalization for HF—as well as the New York Heart Association (NYHA) functional class at follow-up.

### 2.5. Statistical Analysis

Continuous data are presented as median and interquartile range or as mean and standard deviation, as appropriate. Categorical data are shown as absolute and relative frequencies. The *t*-test or the Mann–Whitney U test was used for the comparison between continuous variables, as appropriate. The chi-square test was used for comparing categorical variables. Spearman’s rank correlation coefficient was chosen to evaluate monotonic associations between continuous variables and ordinal or categorical variables without assuming normal distribution. Univariate and multivariate Cox proportional hazard regression analyses were applied to assess predictors of outcomes. A receiver operating characteristic (ROC) analysis was performed to identify cut-offs for predicting the outcome using the Youden index. Kaplan–Meier analysis was used to estimate event-free survival. A *p*-value < 0.05 was considered statistically significant. All analyses were performed using SPSS, version 26 (SPSS, Chicago, IL, USA).

## 3. Results

### 3.1. Patients Population

**Figure 1:**
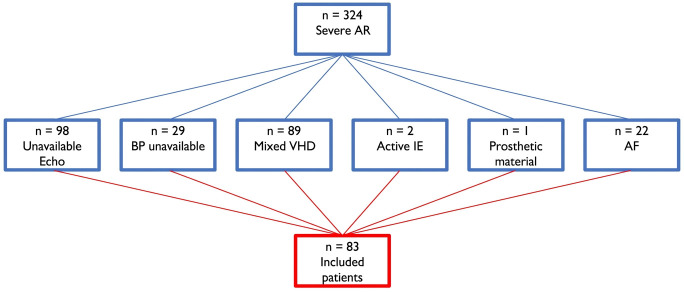
Screening of patients with severe aortic regurgitation based on inclusion and exclusion criteria.

Based on the inclusion criteria, 324 patients with severe AR were identified to be potentially included in the study. A total of 241 patients were excluded based on the aforementioned criteria as shown in Figure 1. A total of 83 patients were finally included in the study, mostly men (62, 74%) with a median age of 71 (IQR: 62–78). Most patients were symptomatic (NYHA II-IV) at time of evaluation (57, 69%) and with preserved EF (>50%) at echocardiography (66, 80%), with a median value of 55 (IQR: 50–57%). However, the mean value of LV GLS was −15 ± 5%. BAV was reported in 15 patients (18%). A total of 28 patients (33%) underwent aortic root or ascending aorta replacement due to dilation, mostly with AV repair (valve sparing aortic root replacement, VSARR) (16, 19%), while a minority underwent a Bentall procedure (12, 14%). Among 52 patients treated with isolated aortic valve surgery, 43 patients received bioprosthetic valves (51%), five patients received mechanical prosthetic valves (6%), and four patients underwent AV repair (5%). Finally, three (3%) patients had significant coronary artery disease treated with coronary artery bypass graft (CABG). The population was divided into two groups according to the composite primary endpoint. Table 1 shows demographic and clinical characteristics of the study population.

### 3.2. Left Ventricular Myocardial Work at Baseline

**Figure 2:**
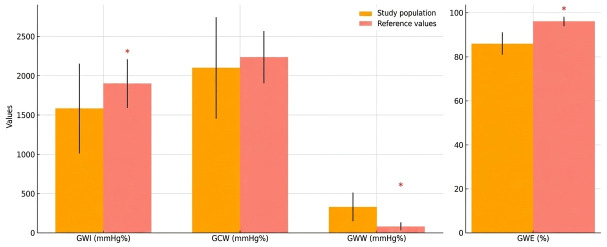
Myocardial work indices in study population (orange) compared to normal reference value derived from NORRE (*European Association of Cardiovascular Imaging Normal Reference Ranges for Echocardiography*) study (red) [25]. Red asterisks indicate statistically significant differences.

The analysis comparing MW parameters in the study population to established reference values revealed significant differences as shown in Table 2 and Figure 2. The mean GWI in the study cohort was 1579.96 ± 568.24 mmHg%, significantly lower than the reference value of 1896 ± 308 mmHg% (*p* < 0.001). Similarly, the mean GCW was 2097.30 ± 645.88 mmHg%, slightly below the reference value of 2232 ± 331 mmHg%, though not statistically significant (*p* = 0.061). In contrast, the mean GWW was 327.95 ± 181.61 mmHg%, markedly higher than the reference value of 78.5 ± 51.26 mmHg% (*p* < 0.001). Additionally, the GWE was 86 (IQR: 81–90)%, significantly lower than the reference median of 96 (IQR: 94–97)% (*p* < 0.001). Furthermore, in absolute terms, 74.7% of patients had a GWI below the reference range, 56.6% had a GCW below the reference range, 95.2% had a GWW above the reference range, and 97.6% had a GWE below the reference range.

Interestingly, when compared to patients with tricuspid aortic valve, we found lower GWE values in patients with BAV, with a statistically significant difference between the groups (88, IQR: 88–90.5 vs. 85, IQR 80–90%, *p* = 0.042).

These findings highlight substantial deviations in myocardial work parameters in the study population, particularly elevated GWW and reduced GWE, suggesting significant myocardial inefficiency and wasted energy, that might be even more evident in BAV related AR.

The correlation analysis revealed significant relationships between several MW indices and echocardiographic parameters of myocardial global contractility (Table 3; see Supplementary Data online, Figure S1). GWI showed a strong negative correlation with GLS (ρ = −0.758, *p* < 0.001) and a moderate positive correlation with LVEF (ρ = 0.520, *p* < 0.001). Similarly, GCW displayed a strong negative correlation with GLS (ρ = −0.753, *p* < 0.001) and a moderate positive correlation with LVEF (ρ = 0.526, *p* < 0.001). In contrast, GWW demonstrated no significant correlation with either GLS (ρ = 0.168, *p* = 0.129) or LVEF (ρ = −0.108, *p* = 0.330). Finally, GWE showed a moderate negative correlation with GLS (ρ = −0.541, *p* < 0.001) and a weak positive correlation with LVEF (ρ = 0.367, *p* < 0.001). These findings highlight the interplay between MW indices and echocardiographic measures of cardiac function.

### 3.3. Patients with Composite Endpoints Versus No Composite Endpoints

The median follow-up duration was 25 (IQR: 15–33) months. Primary composite endpoints occurred in 20 patients during the follow-up (24%), and in particular, six patients (7%) died (five due to cardiovascular causes), and 14 underwent unplanned hospitalization for HF (17%). A total of 39 patients were symptomatic (NYHA class II-IV) at the time of follow-up (46%); 38 had NYHA I (46%), 36 had NYHA II (43%), three had NYHA III (3%) and none had NYHA IV. Patients who developed the primary endpoint were more frequently women (*p* = 0.02) and tended to be older, although this difference did not reach statistical significance. Echocardiographic evaluation revealed higher sPAP values in the primary endpoint group (*p* = 0.022), while LV EF (*p* = 0.079) and left atrial volume (*p* = 0.076) demonstrated a trend toward significance but did not meet the threshold. Regarding MW analysis, patients with primary endpoint occurrence exhibited higher GWW values (*p* = 0.017) and lower GWE values (*p* = 0.013), with no significant differences observed in GWI (*p* = 0.250) or GCW (*p* = 0.341) (Table 1 and Table 4).

Regarding the secondary endpoints, including all-cause deaths and unplanned hospitalizations for HF as individual outcomes, patients who experienced unplanned hospitalization for HF showed a trend toward lower GWE values; however, no statistically significant differences were observed between these groups (see Supplementary Data online, Tables S1 and S2).

### 3.4. Prognostic Analysis

The results of the univariate Cox regression analysis, evaluating the relationship between MW parameters and the composite endpoint, revealed that higher GWE was significantly associated with a reduced risk of the composite endpoint (HR 0.939, 95% CI 0.888–0.993, *p* = 0.028). Conversely, no significant associations were found for GWI (HR 0.999, 95% CI 0.998–1.000, *p* = 0.145) or GCW (HR 0.999, 95% CI 0.999–1.000, *p* = 0.226). GWW showed a trend toward an increased risk with higher values (HR 1.002, 95% CI 1.000–1.005, *p* = 0.069) but did not reach statistical significance (Table 5).

Given the limited number of outcome events, a parsimonious multivariable Cox proportional hazards model was constructed including only clinically relevant covariates (age and NYHA functional class). To further reduce the risk of small-sample bias and overfitting, a penalized Cox regression with Firth correction was additionally performed as sensitivity analysis. In the multivariable Cox model, GWE remained independently associated with the composite endpoint after adjustment for age and NYHA class (HR 0.93, 95% CI 0.87–0.99, *p* = 0.024 in Firth-penalized analysis) (see Supplementary Data online, Table S3).

Regarding the secondary endpoints none of the MW parameters (GWI, GCW, GWW, and GWE) were statistically significant predictors for both all-cause deaths and unplanned hospitalization for HF as single outcomes (see Supplementary Data online, Tables S4 and S5). GWE showed a trend towards a protective effect regarding HF hospitalization, without reaching statistical significance (HR = 0.945, 95% CI 0.884–1.010, *p* = 0.095). The analysis of the relationship between MW indices (GWI, GCW, GWW, and GWE) and NYHA class at follow-up showed no significant correlations. Spearman correlation coefficients were low (ranging from −0.095 to 0.082), with *p*-values greater than 0.4 for all indices (see Supplementary Data online, Table S6 and Figure S2).

**Figure 3:**
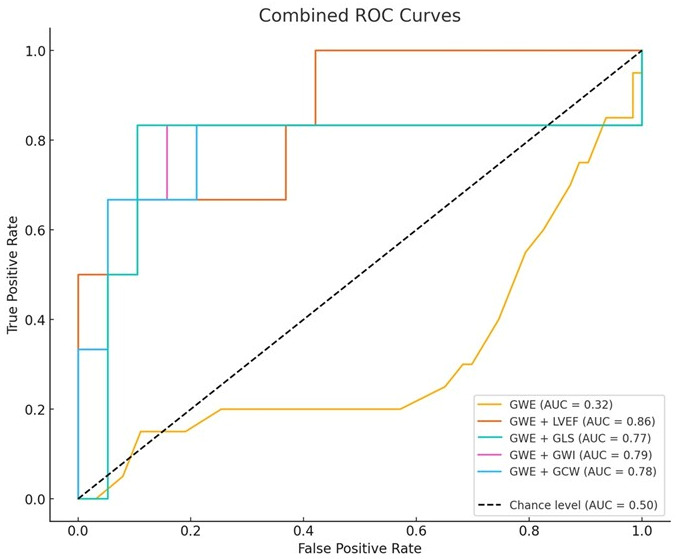
ROC curve for primary composite endpoint (all-cause deaths and unplanned hospitalization for heart failure) of global work efficiency alone (yellow curve) and in combination with other myocardial work indices or left ventricle global systolic function parameters.

The performance of GWE for predicting the primary composite endpoint, assessed through ROC curve analysis, yielded an area under the curve (AUC) of 0.3151 (Figure 3, yellow curve). This suggests that GWE alone has limited predictive ability in identifying patients who developed the primary composite endpoint.

However, the discriminatory power significantly improved when GWE was combined with other variables, as depicted in Figure 3. For instance, combining GWE with LVEF increased the AUC to 0.86, indicating strong predictive performance. Other combinations, such as GWE + GLS (AUC = 0.77) and GWE + GWI (AUC = 0.79), also demonstrated notable improvements compared to GWE alone. These findings underscore the value of integrating complementary variables to enhance the prediction of cardiovascular events. The derived GWE optimal cut-off value for the prediction of the primary composite endpoint using the Youden index was 85.5%.

### 3.5. Survival Analysis

**Figure 4:**
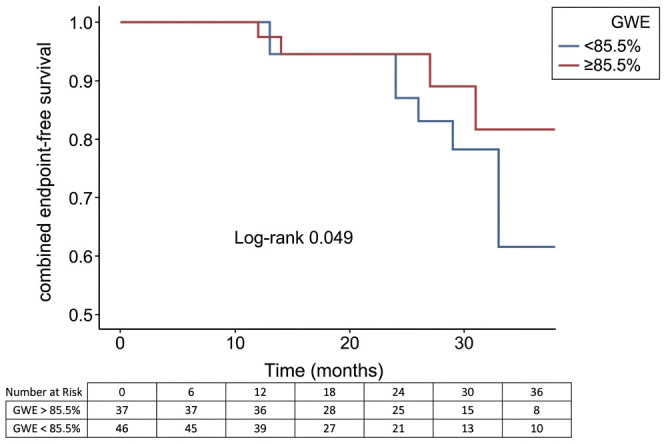
Kaplan–Meier curve for survival free from all-cause deaths and unplanned hospitalization for heart failure according to global work efficiency (GWE). Patients were stratified by GWE > 85.5% versus GWE < 85.5%. Number at risk is shown below the *x*-axis. Comparison between groups was performed using the log-rank test.

The Kaplan–Meier analysis was used to demonstrate that patients with GWE > 85.5% had better prognosis in terms of combined endpoint-free survival, compared to patients with lower values (log-rank = 0.049), as shown in Figure 4.

## 4. Discussion

To the best of our knowledge, this is one of the largest studies evaluating non-invasive echocardiographic myocardial work (MW) in patients with severe aortic regurgitation (AR) undergoing cardiac surgery. The main findings of our study are: (1) patients with severe AR showed significantly reduced global work index (GWI) and global work efficiency (GWE), with markedly increased global wasted work (GWW), compared with normal reference values; (2) patients experiencing adverse cardiovascular events had higher GWW and lower GWE; and (3) higher GWE was independently associated with a reduced risk of the composite endpoint, with an optimal cut-off value of 85.5% for prognostic stratification.

Optimal timing of surgery in chronic severe AR remains challenging. Current guidelines recommend intervention based on symptoms, left ventricular (LV) dilatation, or reduced left ventricular ejection fraction (LVEF) [7,27]. However, several studies have demonstrated that irreversible myocardial damage may develop even in asymptomatic patients with preserved LVEF, leading to worse postoperative outcomes. This highlights the need for more sensitive parameters able to detect early LV dysfunction and guide timely intervention.

Global longitudinal strain (GLS) is a well-established marker of subclinical LV systolic dysfunction and has proven prognostic value in chronic AR [11,12,28,29,30,31]. Nevertheless, GLS is strongly influenced by loading conditions, which is particularly relevant in AR, where chronic volume overload and increased wall stress may mask intrinsic contractile impairment. Myocardial work integrates GLS with non-invasively estimated LV pressure, providing a more load-adjusted assessment of myocardial performance.

In our cohort, MW indices were markedly impaired compared with reference values, reflecting reduced myocardial efficiency and increased wasted energy. GWI, GCW and GWE showed significant correlations with conventional parameters of LV systolic function, such as LVEF and GLS, supporting their physiological relevance. These findings differ partially from those reported by Meucci et al. [32], who observed preserved or increased MW indices in patients with moderate-to-severe AR and preserved LVEF. This discrepancy is likely explained by differences in patient selection. Our study included only patients with severe AR, many of whom were symptomatic and had more advanced myocardial dysfunction, as suggested by lower GLS values. These observations support the concept that MW indices may remain preserved in earlier disease stages as a compensatory mechanism, while progressive LV remodeling and fibrosis eventually lead to reduced myocardial efficiency. To confirm this, in their study Meucci et al. found a post-surgical reduction of LV GWI in 28% of their population, and this was closely associated with worse LV reverse remodeling and could be related to a greater extent of myocardial fibrosis, while patients with preserved GWI showed good LV contractile performance despite impaired values of LV GLS.

Recently, Tan et al. [33] analysed 141 patients with chronic severe AR with preserved LVEF undergoing AV surgery, reporting that lower preoperative GWI (adjusted odds ratio (OR): 0.99; 95% CI: 0.98–1.00; *p* < 0.001) and GCW (adjusted OR: 0.99; 95% CI: 0.99–1.00; *p* < 0.001) were associated with post operative LV disfunction (LVED < 50% at 12 months after intervention) in multivariate analysis. This supports our findings, suggesting that preoperative MW indices are sensitive and load-independent markers of irreversible myocardial damage that could prevent LV contractile recovery even after a successful AV surgery.

Importantly, in our study patients experiencing death or unplanned hospitalization for heart failure exhibited significantly higher GWW and lower GWE. On univariate and multivariate analysis, GWE was associated with a reduced risk of adverse outcomes, and a cut-off value of 85.5% identified patients with better event-free survival. These findings are consistent with previous evidence by D’Andrea et al. [34] showing that GWE reflects the balance between constructive and wasted myocardial work and may serve as a marker of LV contractile reserve. In patients with AR, where a long asymptomatic phase is common, GWE may help identify individuals with early LV decompensation who could benefit from closer surveillance or earlier intervention.

Although GWE alone showed limited discriminative power, its prognostic accuracy markedly improved when combined with other echocardiographic parameters, such as LVEF or GLS. This supports a multiparametric approach to risk stratification, rather than reliance on a single marker.

Curiously, a statistically significant increase in GWW and concomitant reduction in GWE was demonstrated by Toprak et al. [35] in 38 patients with functionally normal bicuspid aortic valve (BAV) and preserved LV systolic function, compared to 44 healthy controls. These MW abnormalities, present even without significant AR or AS, were related to higher arterial stiffness, measured non-invasively by a tonometry system. This could provide fascinating insights into the pathophysiology of AR related to BAV, and to aortic disease in general. Our study involved 15 patients with BAV (18%). However, when compared to patients with tricuspid aortic valve, we found lower GWE values in the latter, with a statistically significant difference between the groups (88, IQR: 88–90.5 vs. 85, IQR 80–90%, *p* = 0.042). It is reasonable to conclude that in the context of BAV there is a complex interplay between valve dysfunction and aortic pathology. Dedicated studies, with a larger population, are warranted to reach unequivocal conclusions.

In recent years, the use of transcatheter aortic valve implantation (TAVI) for pure AR without stenosis has become increasingly adopted [36]. Accordingly, the latest ESC guidelines on VHD have established that TAVI may be considered in patients with severe symptomatic AR who are not eligible for surgery. This should be performed in experienced centers, preferring the use of dedicated devices other than those used for AS, due to technical challenges related to the lack of annular calcification and anchoring issues that lead to a greater risk of valve mispositioning and residual AR [27]. Despite procedural complexities and the implementation requirements of this therapeutic option, the use of the transcatheter approach for the treatment of AR is destined to broaden the therapeutic horizons for this population. Indeed, this will increase the number of treatable patients, including those at the highest surgical risk. Furthermore, the use of MW has proven useful as a diagnostic and prognostic tool in patients with aortic stenosis undergoing TAVI [37,38,39,40]. Recently, Liao et al. [41] conducted a pioneering prospective cohort study involving 43 patients with pure AR undergoing TAVI. Complete echocardiography with MW analysis was performed at baseline and at 1 week, 1 month, 3 months and 6 months post-TAVI. Noninvasive MW analysis proved to be an effective tool for assessing postoperative improvements in LV myocardial function, as GWI and GCW at baseline and 6 months after TAVI were significantly higher in patients exhibiting left ventricular reverse remodeling. These data complement our results in patients who underwent AV surgery, supporting our hypothesis that MW represents a valuable tool for evaluating contractile reserve and predicting outcomes after surgical or percutaneous management of this population.

Several limitations should be acknowledged. The retrospective, single-center design and the relatively small sample size may limit generalizability and statistical power. Follow-up was partly telematic, and repeated echocardiographic evaluations were not available. The use of telephone-based interviews for follow-up constitutes a notable methodological constraint. Despite the high reliability of survival status data, evaluating secondary endpoints like NYHA functional status and heart failure-related admissions via phone increases the risk of recall bias. For the same reasons, precise data on incidence of early and delayed complication are not available. This limitation is particularly pertinent to our retrospective framework and may impact the granularity and accuracy of the observed results.

Nevertheless, this study represents the largest experience of MW assessment in severe AR to date and provides clinically relevant insights that warrant confirmation in larger, prospective, multicenter studies.

## 5. Conclusions

In conclusion, myocardial work analysis, particularly GWE, offers incremental prognostic information in patients with severe AR undergoing surgery. By integrating blood pressure with strain analysis, MW provides a load-adjusted evaluation of LV performance that may improve risk stratification beyond conventional parameters. Future studies are needed to define its role in guiding the timing of intervention and postoperative management in this population.

## Figures and Tables

**Table 1:** Clinical and demographic characteristics of the study population.

	Total Population (*n* = 83)	No Primary Endpoint (*n* = 63)	Primary Endpoint Occurrence (*n* = 20)	*p*-Value
Demographic data				
Age (years)	71 (62; 78)	69 (60; 78)	73 (64; 80)	0.070
**Female sex, *n* (%)**	**21 (25)**	**12 (19)**	**9 (45)**	**0.020**
Weight (Kg)	79 ± 12	79 ± 13	81 ± 10	0.561
BSA (m^2^)	1.95 ± 0.18	1.95 ± 0.18	1.99 ± 0.18	0.438
Clinical data				
DM, *n* (%)	6 (7)	3 (5)	3 (15)	0.248
Hypertension, *n* (%)	57 (69)	42 (67)	15 (80)	0.354
Dyslipidemia, *n* (%)	34 (41)	26 (42)	8 (40)	0.912
Tabagism, *n* (%)	32 (39)	26 (42)	6 (30)	0.444
CCS, *n* (%)	26 (31)	17 (27)	9 (45)	0.071
BAV, *n* (%)	15 (18)	12 (19)	3 (15)	0.939
Symptoms (NYHA II-IV), *n* (%)	57 (69)	44 (70)	13 (45)	0.684
SBP (mmHg)	135 (120; 145)	135 (120; 145)	130 (120; 154)	0.774
DBP (mmHg)	69 ± 12	68 ± 13	73 ± 11	0.097
HR (bpm)	70 ± 12	70 ± 13	71 ± 10	0.807
Laboratory data				
Hb (g/dL)	10.7 (9.9; 12.5)	10.7 (9.9; 12.3)	11 (10; 13)	0.347
Creatinine (mg/dL)	0.96 (0.85; 1.1)	0.93 (0.85; 1.1)	1 (0.8; 1.2)	0.362
AST (UI/L)	22 (16; 29)	22 (16; 29)	20 (14; 27)	0.418
ALT (UI/L)	20 (14; 30)	21 (14; 33)	18 (13; 23)	0.174
Pharmacological data				
ACE-I or ARBs, *n* (%)	46 (59)	35 (56)	11 (55)	0.850
BB, *n* (%)	27 (33)	22 (35)	5 (25)	0.538
Calcium antagonists, *n* (%)	20 (24)	17 (27)	3 (15)	0.386
Loop diuretics, *n* (%)	34 (41)	23 (37)	11(55)	0.086
MRAs, *n* (%)	12 (15)	8 (13)	4 (20)	0.656
Statins, *n* (%)	24 (29)	19 (30)	5 (25)	0.833
Antiplatelets, *n* (%)	26 (31)	20 (32)	6 (30)	0.930
DOACs	6 (7)	4 (6)	2 (10)	0.400

Clinical and demographic characteristics of the study population, divided into two groups based on the primary composite endpoint occurrence. Continue variables expressed as mean value ± standard deviation or as median value with interquartile range, categorical variables expressed as absolute and relative frequence. Statistically significant differences are shown in bold. ACE-I: angiotensin converter enzyme inhibitors; ALT: alanine transaminase; ARBs: angiotensin receptor blockers; AST: aspartate transaminase; BAV: bicuspid aortic valve; BSA: body surface area; CCS: chronic coronary syndrome; DBP: diastolic blood pressure; DM: diabetes mellitus; DOACs: direct oral anti-coagulants; Hb: hemoglobin; HR: heart rate; MRAs: mineralcorticoid receptor antagonists; NYHA: New York Heart Association; SBP: systolic blood pressure.

**Table 2:** Myocardial Work indices in study population compared to normal reference value.

Patameter	Population Value	Reference Value [25]	*p*-Value
**GWI (mmHg%)**	**1579.96 ± 568.24**	**1896 ± 308**	**<0.001**
GCW (mmHg%)	2097.30 ± 645.88	2232 ± 331	0.06
**GWW (mmHg%)**	**327.95 ± 181.61**	**78.5 ± 51.26**	**<0.001**
**GWE (%)**	**86.0 [81.0–90.0]**	**96 [94–97]**	**<0.001**

Myocardial Work indices in study population compared to normal reference value derived from NORRE (*European Association of Cardiovascular Imaging Normal Reference Ranges for Echocardiography*) study [25]. Data expressed as mean value ± standard deviation or as median value with interquartile range. Statistically significant differences are shown in bold. GCW: global constructive work; GWE: global work efficiency; GWI: global work index; GWW: global wasted work.

**Table 3:** Correlation analysis between myocardial work indices and other parameters of left ventricle global systolic function.

Correlation	Spearman’s Coefficient	*p*-Value
**GWI vs. GLS**	**−0.758**	**<0.001**
**GWI vs. LVEF**	**0.520**	**<0.001**
**GCW vs. GLS**	**−0.753**	**<0.001**
**GCW vs. LVEF**	**0.526**	**<0.001**
GWW vs. GLS	0.168	0.129
GWW vs. LVEF	−0.108	0.330
**GWE vs. GLS**	**−0.541**	**<0.001**
**GWE vs. LVEF**	**0.367**	**<0.001**

Statistically significant differences are shown in bold. GCW: global constructive work; GLS: global longitudinal strain; GWE: global work efficiency; GWI: global work index; GWW: global wasted work; LVEF: left ventricle ejection fraction.

**Table 4:** Echocardiographic data from study population, divided into two groups based on primary composite endpoint occurrence.

	Total Population (*n* = 83)	No Primary Endpoint (*n* = 63)	Primary Endpoint Occurence (*n* = 20)	*p*-Value
Echocardiographic data				
LV EDD (mm)	58 (50; 62)	58 (49; 62)	57 (50; 63)	0.924
LV EDD index (mm/mq)	30 (26; 32)	30 (26; 32)	30 (25; 33)	0.953
LV ESD (mm)	35 (30; 40)	34 (30; 40)	39 (30; 43)	0.265
LV ESD index (mm/mq)	19 (15; 22)	18 (15; 21)	20 (14; 23)	0.511
MV E velocity (m/s)	0.72 ± 0.26	0.74 ± 0.26	0.68 ± 0.24	0.464
MV DecT (ms)	203 (178; 235)	212 (181; 237)	192 (165; 231)	0.153
LV EDV (mL)	155 ± 50	157 ± 51	147 ± 50	0.475
LV EDV index (mL/mq)	82 ± 24	83 ± 24	79 ± 24	0.563
LV EF (%)	55 (50; 57)	55 (50; 60)	52(40; 56)	0.079
LV EF < 50%, *n* (%)	17 (20)	10 (16)	7 (35)	0.126
LAV (mL)	78 (61; 100)	74 (61; 90)	101 (55; 126)	0.076
TAPSE (mm)	23 (20; 26)	24 (20; 27)	22 (18; 26)	0.835
TDI RVs’ (m/s)	0.13 (0.11; 0.15)	0.13 (0.11; 0.15)	0.13 (0.11; 0.17)	0.801
**sPAP (mmHg)**	**30 (25; 35)**	**25 (25; 34)**	**30 (28; 37)**	**0.022**
Aortic root (mm)	40 ± 7	40 ± 7	40 ± 6	0.539
Ascending aorta (mm)	43 ± 8	42 ± 8	45 ± 9	0.142
Aortic arch (mm)	30 ± 5	31 ± 5	30 ± 4	0.539
IVC (mm)	18(16; 21)	18 (16; 21)	18 (16; 21)	0.835
GLS-4CH (%)	−15 ± 4	−16 ± 4	−14 ± 5	0.130
GLS-2CH (%)	−15 ± 4	−16 ± 4	−15 ± 5	0.590
GLS-3CH (%)	−16 ± 5	−16 ± 5	−14 ± 5	0.102
GLS-avg (%)	−15 ± 5	−15 ± 5	−14 ± 5	0.293
GWI (mmHg%)	1580 ± 568	1621 ± 565	1450 ± 567	0.250
GCW (mmHg%)	2097 ± 646	2136 ± 649	1977 ± 638	0.341
**GWW (mmHg%)**	**327 ± 182**	**295 ± 154**	**432 ± 224**	**0.017**
**GWE (%)**	**86 (81; 90)**	**88 (82; 91)**	**82 (77; 86)**	**0.013**

Variables expressed as mean value ± standard deviation or as median value with interquartile range. Statistically significant differences are shown in bold. 2CH: 2-chambers view; 3CH: 3-chambers view; 4CH: 4-chambers view; avg: average; DecT: deceleration time; EDD: end diastolic diameter; EDV: end diastolic volume; EF: ejection fraction; ESD: end systolic diameter; GCW: global constructive work; GLS: global longitudinal strain; GWE: global work efficiency; GWI: global work index; GWW: global wasted work; IVC: inferior vena cava; LAV: left atrium volume; LV: left ventricle; MV: mitral valve; RV: right ventricle; sPAP: systolic pulmonary artery pressure; TAPSE: tricuspid annulus plane systolic excursion; TDI: tissue Doppler.

**Table 5:** Cox regression analysis regarding the relationship between myocardial work parameters and the primary composite endpoint.

Parameter	HR (95% CI)	*p*-Value
GWI (mmHg%)	0.999 (0.998–1)	0.145
GCW (mmHg%)	0.999 (0.999–1)	0.226
GWW (mmHg%)	1.002 (1–1.005)	0.069
**GWE (%)**	**0.939 (0.888–0.993)**	**0.028**

Cox regression analysis regarding the relationship between myocardial work parameters and the primary composite endpoint (all-cause deaths and unplanned hospitalization for heart failure). Statistically significant differenvces are shown in bold. CI: confidence interval; GCW: global constructive work; GWE: global work efficiency; GWI: global work index; GWW: global wasted eork; HR: hazard ratio.

## Data Availability

The data supporting the findings of this study are available from the corresponding author upon reasonable request.

## Supplementary Materials

The following supporting information can be downloaded at: https://www.mdpi.com/article/10.3390/diagnostics16111655/s1, Figure S1. Scatter plots represent the correlation between Myocardial Work indices and other parameters of left ventricle global systolic function. Figure S2. Scattered plots that represent the relationship between Myocardial Work indices and NYHA class. Table S1. Comparison of Speckle Tracking Echocardiography e Myocardial Work parameters in study population, divided into two groups based on the occurrence (group 1) or not (group 0) of secondary endpoint of all-cause death. Variables expressed as mean value ± standard deviation or as median value with interquartile range. Table S2. Comparison of Speckle Tracking Echocardiography e Myocardial Work parameters in study population, divided into two groups based on the occurrence (group 1) or not (group 0) of secondary endpoint of unplanned hospitalization for heart failure. Variables expressed as mean value ± standard deviation or as median value with interquartile range. Table S3. Sensitivity analysis using Firth-penalized Cox proportional hazards regression. Table S4. Cox regression analysis regarding the relationship between Myocardial Work Parameters and the secondary endpoint of all-cause deaths. Table S5. Cox regression analysis regarding the relationship between Myocardial Work Parameters and the secondary endpoint of unplanned hospitalization for heart failure. Table S6. Correlation analysis between Myocardial Work indices and NYHA (New York Heart Association) class.
