# Supplementary material for: Additional Role of Myocardial Work in Prognostic Stratification of Patients with Severe Aortic Regurgitation Undergoing Aortic Valve Surgery
Source: Diagnostics (Basel). 2026 May 27;16(11):1655. doi: 10.3390/diagnostics16111655 (PMC13257256; doi:10.3390/diagnostics16111655)
Supplement: Supplementary file 1 [file diagnostics-16-01655-s001.zip › diagnostics-4256382-supplementary.pdf]

Additional Role of Myocardial Work in Prognostic Stratification of Patients with Severe Aortic Regurgitation Undergoing Aortic Valve Surgery

Giulia Elena Mandoli, Gerardo Elia Del Vecchio, Nicolò Ghionzoli, Luca Corda, Pamela Tartaglia, Andrea Stefanini, Maria Concetta Pastore, Francesco Morrone, Marta Focardi, Matteo Lisi, Antonello D'andrea, Matteo Cameli

SUPPLEMENTARY DATA

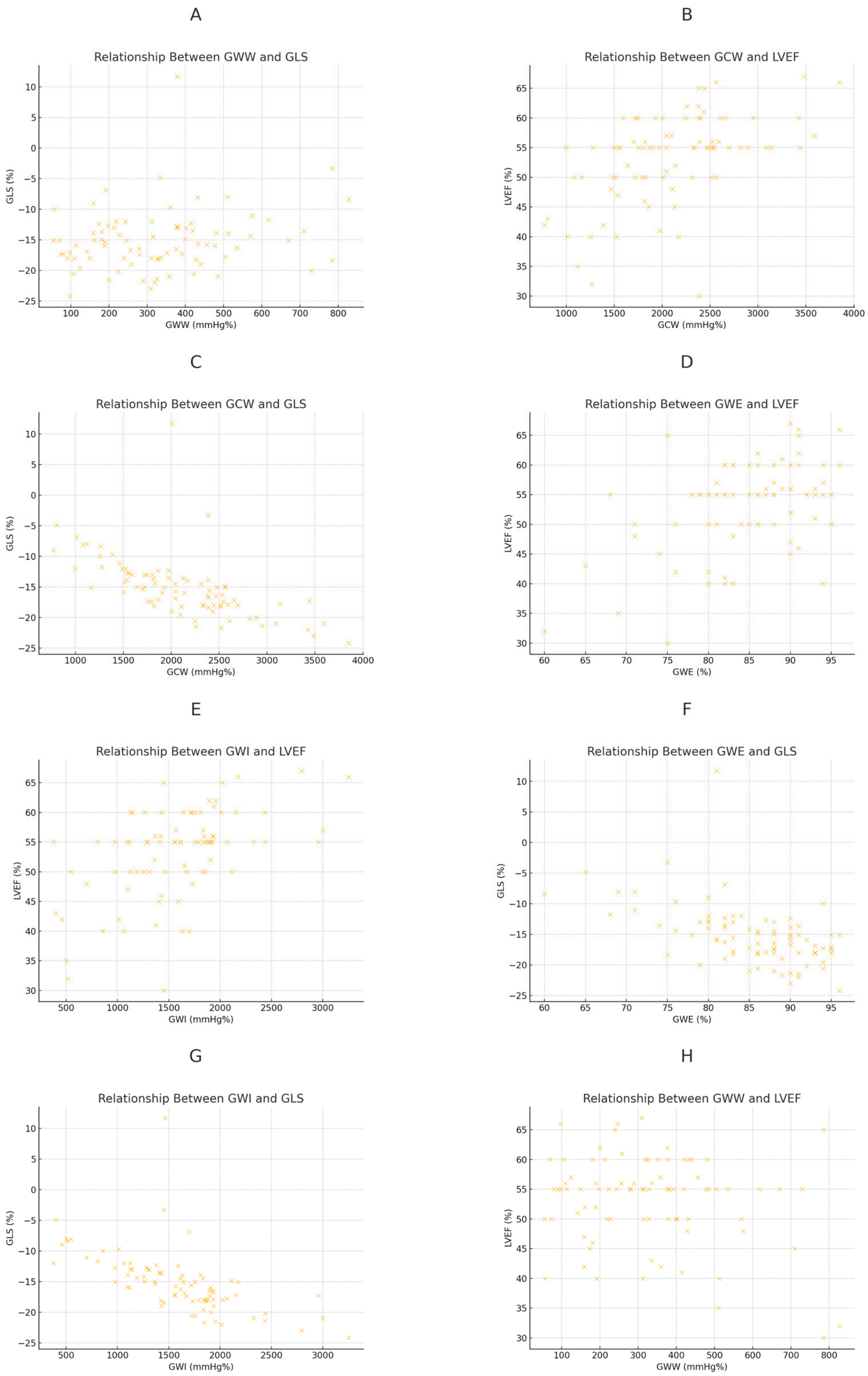

Figure S1. Scatter plots represent the correlation between Myocardial Work indices and other parameters of left ventricle global systolic function. GCW: Global Constructive Work; GLS:

Global Longitudinal Strain del VS; GWE: Global Work Efficiency; GWI: Global Work Index; GWW: Global Wasted Work; LVEF: Left Ventricle Ejection Fraction.

**Parameter**	**Group 0**	**Group 1**	***p-value***
GLS-4CH	-15.30 ± 4.45	-13.15 ± 4.62	0.315
GLS-2CH	-15.45 ± 4.47	-15.60 ± 3.40	0.922
GLS-3CH	-15.61 ± 4.91	-15.75 ± 4.48	0.943
GLS	-15.18 ± 5.13	-14.87 ± 3.53	0.847
GWI (myocardial work)	1582.64 ± 573.84	1545.67 ± 537.40	0.877
GCW (myocardial work)	2099.36 ± 659.12	2070.83 ± 486.81	0.897
GWW (myocardial work)	317.48 ± 173.59	462.33 ± 243.93	0.209
GWE (myocardial work)	87.00 [81.00-90.00]	82.50 [80.50-85.25]	0.396

Table S1. Comparison of Speckle Tracking Echocardiography e Myocardial Work parameters in study population, divided into two groups based on the occurrence (group 1) or not (group 0) of secondary endpoint of all-cause deaths. Variables expressed as mean value ± standard deviation or as median value with interquartile range. 2CH: 2-chambers view; 3CH: 3-chambers view; 4CH: 4-chambers view; avg: Average; GLS: Global Longitudinal Strain; GCW: Global Constructive Work; GWE: Global Work Efficiency; GWI: Global Work Index; GWW: Global Wasted Work.

**Parameter**	**Group 0**	**Group 1**	***p-value***
GLS-4CH	-15.43 ± 4.09	-13.74 ± 6.00	0.329
GLS-2CH	-15.62 ± 4.05	-14.65 ± 5.87	0.561
GLS-3CH	-16.11 ± 4.64	-13.21 ± 5.33	0.075
GLS	-15.43 ± 4.92	-13.81 ± 5.45	0.319
GWI (myocardial work)	1614.49 ± 561.07	1409.79 ± 593.82	0.251
GCW (myocardial work)	2129.94 ± 633.66	1936.43 ± 705.34	0.354
GWW (myocardial work)	309.42 ± 167.83	419.29 ± 223.36	0.100
GWE (myocardial work)	88.00 [82.00-90.00]	81.50 [76.75-84.50]	0.068

Table S2. Comparison of Speckle Tracking Echocardiography e Myocardial Work parameters in study population, divided into two groups based on the occurrence (group 1) or not (group 0) of secondary endpoint of unplanned hospitalization for heart failure. Variables expressed as mean value ± standard deviation or as median value with interquartile range. 2CH: 2-chambers view; 3CH: 3-chambers view; 4CH: 4-chambers view; avg: Average; GLS: Global Longitudinal Strain; GCW: Global Constructive Work; GWE: Global Work Efficiency; GWI: Global Work Index; GWW: Global Wasted Work.

**Variable**	**Hazard Ratio (HR)**	**95% CI**	**p-value**
GWE	0.93	0.88–0.99	0.024
Age	1.01	0.97–1.07	0.583
NYHA functional class	1.61	0.75–3.41	0.216

Table S3. Sensitivity analysis using Firth-penalized Cox proportional hazards regression. Number of patients included: 79; Number of events: 19. CI: confidence interval; GWE: global work efficiency; HR: hazard ratio; NYHA: New York Heart Association.

**Parameter**	**HR (95% CI)**	***p-value***
GWI (mmHg%)	0.999 (0.997-1.001)	0.512
GCW (mmHg%)	1 (0.998-1.001)	0.703
GWW (mmHg%)	1.003 (0.998-1.008)	0.199
GWE (%)	0.928 (0.835-1.032)	0.928

Table S4. Cox regression analysis regarding the relationship between Myocardial Work Parameters and the secondary endpoint of all-cause deaths. CI: Confidence Interval; GCW: Global Constructive Work; GWE: Global Work Efficiency; GWI: Global Work Index; GWW: Global Wasted Work; HR: Hazard Ratio

**Parameter**	**HR (95% CI)**	***p-value***
GWI (mmHg%)	0.999 (0.998-1)	0.180
GCW (mmHg%)	0.999 (0.998-1)	0.268
GWW (mmHg%)	1.002 (0.999-1.005)	0.196
GWE (%)	0.945 (0.884-1.010)	0.095

Table S5. Cox regression analysis regarding the relationship between Myocardial Work Parameters and the secondary endpoint of unplanned hospitalization for heart failure. CI: Confidence Interval; GCW: Global Constructive Work; GWE: Global Work Efficiency; GWI: Global Work Index; GWW: Global Wasted Work; HR: Hazard Ratio

**Parameter**	**Spearman's Coefficient**	**p-value**
GWI (mmHg%)	-0.066	0.566
GCW (mmHg%)	-0.075	0.519
GWW (mmHg%)	0.082	0.477
GWE (%)	-0.095	0.411

Table S6. Correlation analysis between Myocardial Work indices and NYHA (New York Heart Association) class. GCW: Global Constructive Work; GWE: Global Work Efficiency; GWI: Global Work Index; GWW: Global Wasted Work

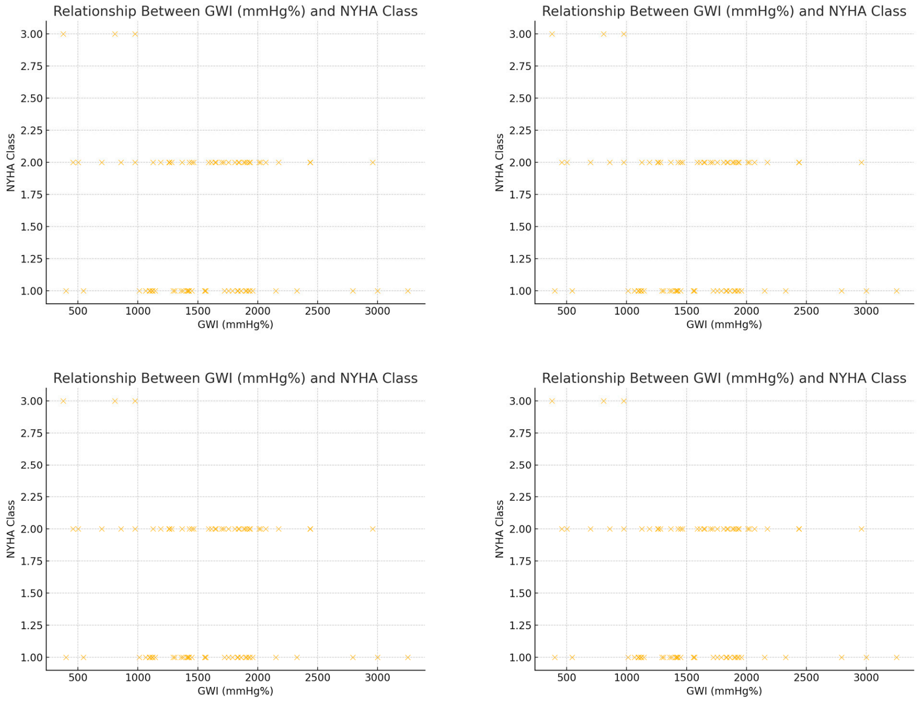

Figure S2. Scattered plots that represent the relationship between Myocardial Work indices and NYHA class. GCW: Global Constructive Work; GWE: Global Work Efficiency; GWI: Global Work Index; GWW: Global Wasted Work; NYHA: New York Heart Association.
